# The Water Insecurity Experiences (WISE) Scales are suitable for use in high-income settings: findings from cognitive interviews and nationally representative surveys

**DOI:** 10.21203/rs.3.rs-6917162/v1

**Published:** 2025-06-23

**Authors:** Sera L. Young, Joshua D. Miller, Indira Bose, Shalean Collins, Sarah Danaj, Boris Kazakov, Ben Pauli, Aleksandra Ravnachka, Adam Ritchie, Kelsey Rydland, Benjamin Sefcovic, Chad Staddon, Sophia Staddon, Jaynie Vonk, Edward A. Frongillo

**Affiliations:** Northwestern University; University of North Carolina at Chapel Hill; London School of Hygiene & Tropical Medicine; Tulane University; Kettering University; Bulgarian Academy of Sciences; Kettering University; Neofit Rilski, South-West University of Blagoevgrad; Kettering University; University Libraries, Northwestern University; Kettering University; University of the West of England; Independent Scholar; Oxfam; University of South Carolina

**Keywords:** high-income countries, low- and middle-income countries, measurement equivalence, scale development, validation, water insecurity

## Abstract

**Background:**

The Water Insecurity Experiences Scales are validated tools for reliably and comparably assessing experiences with water access and use in low- and middle-income countries. Although theoretically applicable in high-income countries, their performance in these settings has not been assessed. This study therefore examined whether the Water Insecurity Experiences Scales function similarly in high-income countries, and if they generated measures comparable to those in low- and middle-income countries.

**Methods:**

We conducted cognitive interviews with 73 adults from 4 high-income countries (Bulgaria, the Netherlands, the United Kingdom, and the United States) to assess whether participants understood the items in the Individual Water Insecurity Experiences Scale as intended. We then used nationally representative Gallup World Poll data from two high-income countries (Australia, the United States) and three low- and middle-income countries (Bangladesh, Brazil, and Uganda) to evaluate internal consistency, unidimensionality, and measurement invariance (n = 4,928).Construct validity was assessed by testing hypothesized associations between water insecurity scores and wealth, household size, self-reported stress, and satisfaction with water quality within Australia and the United States.

**Results:**

Cognitive interviews revealed no major issues with item translation or comprehension, supporting construct equivalence. The prevalence of moderate-to-high water insecurity was low in Australia (3.7%) and the United States (1.0%). In both countries, the scale was internally consistent, conformed to the unidimensional structure, and demonstrated good model fit based on criteria established *a priori*. Configural and scalar measurement invariance were supported across all five countries. As for validity, water insecurity scores were associated with different sociodemographic characteristics (wealth, household size), self-reported stress, and satisfaction with water quality in the directions hypothesized. For example, the percentage of participants with moderate-to-high water insecurity reporting stress during the previous day or water quality dissatisfaction was 1.80 times (95% CI: 1.50, 2.17) and 4.12 times (95% CI: 2.87, 5.93) higher, respectively, than among those with no-to-mild water insecurity.

**Conclusions:**

The Individual Water Insecurity Experiences Scale performs well in high-income countries and yields cross-country comparable measures, supporting its use for global monitoring of water insecurity.

## INTRODUCTION

Water insecurity, the inability to reliably access enough water of sufficient quality for domestic uses (1), is a widespread challenge. Water issues are expected to become more common and severe globally due to climate change, population growth, and rising resource demands (2–4). The consequences of water insecurity are far-reaching, contributing to poor physical (5, 6) and mental health (7), food insecurity and nutrition-related diseases (8, 9), increased risk of violence (10), and reduced economic opportunities (11). Accurate measurement of water insecurity is thus essential for understanding its magnitude and guiding strategies to advance universal water access and improve public health.

Global water monitoring has primarily relied on “provider-side” indicators, such as per capita freshwater availability or the proportion of households using improved water sources (3, 12). While useful for assessing supply and infrastructure, these measures do not capture whether water services meet users’ needs. As such, there is growing recognition of the value of complementary experiential “user-side” metrics that assess whether people can access sufficient water for daily life (8, 13, 14). The Water Insecurity Experiences (WISE) Scales are among the most widely used. These include the 12-item Household Water Insecurity Experiences (HWISE) and Individual Water Insecurity Experiences (IWISE) Scales (17, 18) as well as abbreviated four-item versions of each (19, 20). The WISE Scales query about experiences with water for drinking, hygiene, and other domestic uses that are theorized to be universal (Supplementary Table 1). Items in the IWISE Scale mirror those in the HWISE Scale but are phrased to reflect individual, rather than household, experiences (21), enabling disaggregation by sociodemographic characteristics, including gender, age, and education (22, 23).

The WISE Scales were developed with global comparability as a primary goal. Tools intended for cross-country monitoring must accurately capture the construct of interest in diverse contexts and function equivalently across settings (15). To meet these criteria, the WISE Scales were developed using data collected in settings selected to maximize heterogeneity in climate, water infrastructure, and population density (16–20). The 12-item HWISE Scale was first validated in 28 sites across 23 low- and middle-income countries (LMIC) (17) while the IWISE Scale was originally validated using nationally representative Gallup World Poll data from 31 LMICs (18).

**Figure 1:**
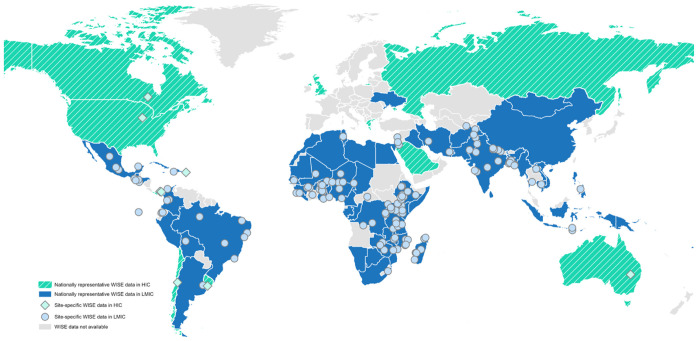
The WISE Scales have been administered globally. As of mid-2025, nationally representative data collection is ongoing or has been completed for 80 countries (shaded) and site-specific data is ongoing or has been completed in at least 154 sites (diamonds and circles).

Since their development, the WISE Scales have been implemented by more than 100 organizations – from local NGOs to United Nations agencies – for a range of purposes, including global monitoring (e.g., 21,22), impact evaluation (e.g., 6,23), advocacy (e.g., 24,25), and understanding the health consequences of water insecurity (e.g., 26–29). Most applications have occurred in LMICs (30, Fig. 1). For example, the Gallup World Poll has included the IWISE Scale in 80 countries across its 2020, 2022, and 2025 rounds, 72 of which are LMICs. It has also been implemented by UNICEF in Mongolia, in the 2020–2021 MICS-Plus survey (77); in Mozambique, in the 2022–2023 round of the Demographic and Health Survey (78); in Tonga in the wake of the 2022 volcanic eruptions (79); and in Mexico, in the National Health and Nutrition Survey (ENSANUT) annually since 2021 (72, 80). The WISE Scales have also been used for site-specific data collection (Fig. 1, diamonds and circles), which has similarly been concentrated in LMICs.

Although the WISE Scales were designed for global use, their application in high-income countries (HICs) has been limited. It is, however, expanding. The Gallup World Poll collected nationally representative IWISE data from the United States and Australia in 2022, with additional HICs – Canada, Greece, Israel, Russia, Ukraine, and the United Kingdom – scheduled for survey in 2025 (Fig. 1, turquoise shading). Site-specific studies using the WISE Scales have also been conducted in a handful of HICs, including Canada (31) and Australia (24). The validity and cross-contextual equivalence of the WISE Scales in HICs, however, have not yet been formally assessed.

Understanding how water insecurity manifests in high-income settings – and whether it can be meaningfully compared to experiences in LMICs – is critical for advancing equity in global monitoring and resource allocation. While water insecurity has traditionally been viewed as a challenge confined to LMICs (32), growing evidence highlights its significance for health and well-being in HICs as well (33–42). To address this gap, we therefore sought to evaluate the performance of the IWISE Scale in HICs. Specifically, we examined whether the IWISE Scale provides valid, reliable, and comparable measures of water insecurity across countries with differing World Bank income classification. We first conducted cognitive interviews with participants in four HICs – Bulgaria, the Netherlands, the United Kingdom, and the United States – to assess whether items were understood as intended. We then used nationally representative data from the United States, Australia, Bangladesh, Brazil, and Uganda to evaluate the scale’s internal consistency, unidimensionality, and measurement invariance across diverse contexts. To further assess construct validity, we tested whether IWISE scores were associated with factors, including household wealth, household size, self-reported stress, and satisfaction with water quality.

## METHODS

### Cognitive interviews

Cognitive interviews are a qualitative technique for understanding if items are understood by respondents as intended (43). In this study, local co-authors conducted cognitive interviews on the 12 IWISE Scale items using a one-year recall period. Interviews were carried out in five sites across four HICs: Bulgaria, the Netherlands, the United Kingdom, and the United States (Flint, Michigan and New Orleans, Louisiana). These sites were selected based on the presence of collaborating researchers familiar with the WISE Scales who had interest in the project and availability to support data collection.

Within each site, participants were selected using various convenience sampling strategies (e.g., snowball sampling, online outreach). To ensure a diversity of perspectives, the protocol encouraged recruitment of approximately eight men and eight women across three age groups: young adults (18–30 years), middle-aged adults (31–55 years), and older adults (> 55 years). There were no exclusion criteria.

Where necessary, site leads translated the items from English to the local language (Bulgarian or Dutch) prior to data collection. Interviews began with an explanation of the purpose of the study: to understand if a set of questions about experiences with water are appropriate and understandable. Interviewers read aloud each IWISE Scale item and asked participants to rephrase the question in their own words, then provide a response. Response options included “never”; “in one or two months of the year”; “in some but not every month of the year”; and “in almost every month of the year”. If participants responded with “I do not know” or “not applicable”, interviewers probed to understand why. Respondents were also asked to explain how they arrived at the reported frequency. Basic sociodemographic information (e.g., age, gender, ethnicity) was collected at the end of the interview.

Interviews lasted approximately 30 minutes. Participants were remunerated between $20 to $50 (or the local equivalent thereof) for their participation.

### Qualitative data analysis

Each interviewer or interviewer team summarized the cognitive interviews, noting any difficulties participants experienced with item translation, comprehension, or response. They also documented whether these challenges varied by respondent gender or age. Based on these observations, interviewers were asked to provide recommendations for improving the phrasing of the IWISE Scale items.

### Survey data collection

To assess the performance of the IWISE Scale in HICs and determine whether it produces scores that are comparable to those from LMICs, we analyzed data from five nationally representative surveys conducted through the Gallup World Poll (44). Two of these datasets were from the two HICs in which IWISE data collection has been completed: the United States (n = 1,003) and Australia (n = 1,000). To make cross-country comparisons with these two datasets, we sought to identify three LMICs that varied by geographic region (Asia, Latin America, and Africa) and national income level, based on the World Bank’s 2022 fiscal year classifications. Using these criteria, we selected Bangladesh (n = 1,009; lower-middle income), Brazil (n = 1,003; upper-middle income), and Uganda (n = 1,000; low-income).

The Gallup World Poll is an annual cross-sectional survey administered to non-institutionalized individuals aged 15 years and older. Full methodological details are described elsewhere (18, 45). Briefly, the Gallup World Poll uses stratified, probability-based sampling strategies to ensure national representativeness and applies sampling weights to account for design effects and non-response. Trained in-country partners conducted telephone interviews in 2020 (Bangladesh, Brazil, Uganda) and 2022 (USA, Australia) using standardized Gallup World Poll protocols.

Wealth was assessed using two measures. Perceived income adequacy was assessed by asking participants if they felt they were “living comfortably on present income”, “getting by on present income”, “finding it difficult on present income”, or “finding it very difficult on present income”. To improve cross-country comparability, responses were dichotomized as either having any difficulty getting by on present income or not (21). Relative income was based on per capita household income, calculated by Gallup from respondents’ reported monthly household incomes and categorized into quintiles (45).

Sociodemographic information was collected using standard Gallup World Poll procedures. Respondent gender was recorded by interviewers as man or woman. Household size was calculated as the total number of household members under and over age 15. Marital status was collapsed into three categories: never married; married or partnered; and divorced, separated, or widowed. Educational attainment was grouped into three categories: elementary (≤ 8 years of education), secondary (9–15 years), and tertiary (≥ 16 years or 4 + years beyond high school).

Two additional variables were included to assess construct validity. Dissatisfaction with local water quality was measured using the question, “In your city or area where you live, are you satisfied or dissatisfied with the quality of water?” Self-reported stress was assessed using the question, “Did you experience stress during a lot of the day yesterday?”

### Survey data analysis

To assess the performance of the IWISE Scale in HICs and its comparability to LMICs, we conducted four types of statistical analyses. Analyses were restricted to individuals with complete data for all IWISE items; no imputation was performed. Few respondents were excluded due to missing responses: 2 in the United States (for an analytic sample of 1,001), 9 in Australia (n = 991), 2 in Bangladesh (n = 1,007), 13 in Brazil (n = 990), and 61 in Uganda (n = 939).

To test for internal consistency – the extent to which items in a scale covary relative to their sum score (46–48) – we calculated Cronbach’s alpha for each country. While a value of 0.70 is typically considered acceptable, a threshold of 0.80 is preferred for establishing strong psychometric quality (48, 49).

We then evaluated whether the IWISE Scale retained its unidimensional structure in each setting. Previous WISE validation studies assessed unidimensionality using confirmatory factor analysis with an independent cluster model (17–20). We therefore conducted separate confirmatory factor analyses for each of the five study countries using Mplus version 8. The 12 IWISE Scale items were treated as categorical variables and estimated using the weighted least square mean- and variance-adjusted (WLSMV) estimator. Model fit was assessed using standard indices: root mean square error of approximation (RMSEA and the upper bound of its 90% confidence interval ≤ 0.05), comparative fit index (CFI > 0.95), Tucker-Lewis index (TLI > 0.95), and standardized root mean square residual (SRMR < 0.08). Items were considered to be related to the latent construct if standardized factor loadings were ≥ 0.70.

Third, we assessed measurement invariance, which examines whether a scale comparably measures the same underlying construct across groups (50). This step is critical to ensure that cross-country differences in IWISE scores reflect true variation in water insecurity rather than differences in how respondents interpret the items. We tested both configural and scalar invariance. Configural invariance assesses whether the same factor structure is present across groups (i.e., the same items load onto the same latent construct), while scalar invariance adds the constraint that item thresholds are equal across groups. Metric invariance was not tested, as it cannot be identified with categorical responses with the selected estimator; if scalar invariance is supported, however, metric invariance can be assumed (51). We performed multi-group confirmatory factor analyses in Mplus using the WLSMV estimator, with country as the group variable. We ran two types of invariance tests: (1) a global model simultaneously comparing all five countries, and (2) pairwise models separately comparing each HIC (United States and Australia) to each LMIC (Bangladesh, Brazil, and Uganda). Model fit was evaluated using standard criteria for RMSEA, CFI, TLI, and SRMR. Changes in fit indices (ΔCFI ≤ 0.01 and ΔRMSEA ≤ 0.015) were used to assess scalar invariance.

Finally, we assessed construct validity by testing whether the IWISE Scale could distinguish between subgroups as theoretically expected. We examined associations between sociodemographic factors – perceived income adequacy, relative income, and household size – and water insecurity. We ran separate linear regressions for each predictor, adjusting for country and incorporating survey weights to account for the complex sampling design. Based on prior research (52, 53), we hypothesized that IWISE scores would be higher in households with lower relative income, those reporting financial hardship, and those with more members.

To further evaluate the validity of the IWISE Scale, we examined the associations between experiential water insecurity and both self-reported stress in the prior day and dissatisfaction with water quality, adjusting for country and the complex sampling strategy. For each outcome, we estimated two Poisson regression models: one treating water insecurity as a continuous variable (IWISE score), and another using a binary indicator of moderate-to-high water insecurity (IWISE score ≥ 12) (54). Results are reported as prevalence ratios with 95% confidence intervals. We hypothesized that higher IWISE Scores would be associated with greater experiences of self-reported stress and water quality dissatisfaction.

### Human subjects approval

The qualitative component of this study was approved by Northwestern University’s Institutional Review Board (STU00213587) and determined as exempt by the Institutional Review Boards at Tulane University and Kettering University. Verbal informed consent was obtained from all participants prior to conducting cognitive interviews.

Survey data were collected by Gallup as part of the Gallup World Poll, following their standard protocols for participant recruitment and informed consent. Analyses presented here are based on deidentified data provided by Gallup; the authors of this paper were not involved in the original data collection or consent procedures.

## RESULTS

### Cognitive interview sample

To understand if IWISE items were understood similarly and as intended across HIC settings, we conducted cognitive interviews with 73 individuals across five sites (Table 1). Approximately half (42/73) of the participants were women, and ages ranged from 18 to 89 years. Most participants were nationals of the country in which they were interviewed; each site included a small number of participants who were ethnic minorities (data not shown).

### Construct equivalence

Overall, participants found the IWISE Scale items to be appropriate and relevant. Items were generally well understood and answerable. In the two sites where English was not the primary language (Bulgaria and the Netherlands), no difficulties with item translation were reported.

Across the five sites, respondents reported consistent interpretations of the items that aligned with their intended meaning. Further, all items were considered applicable and answerable, although a few participants initially struggled to respond to items about situations they had not personally experienced. For example, participants in Bulgaria noted that drinking water was always available; although they had no direct experience with scarcity, they understood what the items related to this issue were asking. In Flint, Michigan, some participants were uncertain whether to consider tap or purchased bottled water when responding. For instance, participants at this site who reported that they “almost always” had enough water to drink were typically referring to bottled rather than tap water, with one respondent explaining that “if stores are out of water, I have no water at all for drinking.”

Interviewers did not observe differences in the ability of participants to answer the items by gender or age. Interestingly, though, responses in Flint, Michigan, revealed gendered patterns in how water issues were experienced. Women often emphasized household-level impacts, such as the effect of water insecurity on family members’ well-being or on daily routines. In contrast, men were more likely to highlight financial strain or concerns about maintaining independence (e.g., not needing assistance to access water).

Most suggestions for improving the items focused on adapting terminology or examples to better reflect the local context. For instance, in the United Kingdom, it was suggested that the more colloquial phrase “pissed off” might be better understand than “angry”. Minor adaptations like these are consistent with guidance provided in the WISE Manual, which encourages local adaptation while preserving the intent of the item (55). There was also a recommendation to develop a skip pattern to reduce burden on respondents not experiencing water-related issues, an option currently being explored.

Finally, some participants expressed dissatisfaction with the one-year recall period, wanting to discuss water experiences that occurred further in the past, such as those during a particular hurricane or the Flint Water Crisis. While a 12-month recall is appropriate for global monitoring, the IWISE Scale is designed to accommodate shorter or alternative recall periods depending on study objectives. Guidance on recall period selection is also available in the WISE Manual (55).

### Gallup World Poll analytic sample

After establishing that the items were understood as intended, we conducted quantitative analyses using survey data from five countries included in the Gallup World Poll (Table 2). Participants from the two HICs (the United States and Australia) were generally older and had more years of formal education than those in Bangladesh, Brazil, and Uganda. A lower percentage of respondents in the HICs reported difficulty getting by on their present income relative to those in the LMICs. The prevalence of moderate-to-high water insecurity was also higher in the LMICs compared to the HICs.

### Internal consistency

The IWISE Scales demonstrated high internal consistency across countries, with Cronbach’s alpha values exceeding 0.83 in each (Table 3). These values suggest high interrelatedness among the items.

### Dimensionality

Confirmatory factor analyses supported the unidimensional structure of the IWISE Scale across all five countries. At the country level, items had high factor loadings and most model fit statistics met the *a priori* criteria (Table 3). The only exceptions were two RMSEA-related values, which slightly exceeded conventional thresholds in two LMICs. This suggests that the scale functioned well within each country and are consistent with findings from prior validation studies of both the HWISE and IWISE Scales (17, 18).

### Measurement invariance

We investigated whether the IWISE Scale functioned equivalently across countries using multi-group confirmatory factor analyses. Fit indices met or closely approached accepted thresholds, supporting both configural and scalar invariance across the five countries (Table 4). These results indicate that the scale measures the same underlying construct across settings, allowing for cross-country comparisons. Pairwise comparisons between each HIC (United States and Australia) and each LMIC (Bangladesh, Brazil, and Uganda) yielded similarly acceptable fit statistics (Supplementary Tables 2–8), indicating consistent measurement properties across diverse contexts.

### Validity

A key indicator of construct validity is a scale’s ability to distinguish between groups expected to differ. We therefore explored if IWISE scores varied by household income (measured two ways) and household size within the United States and Australia.

**Figure 2:**
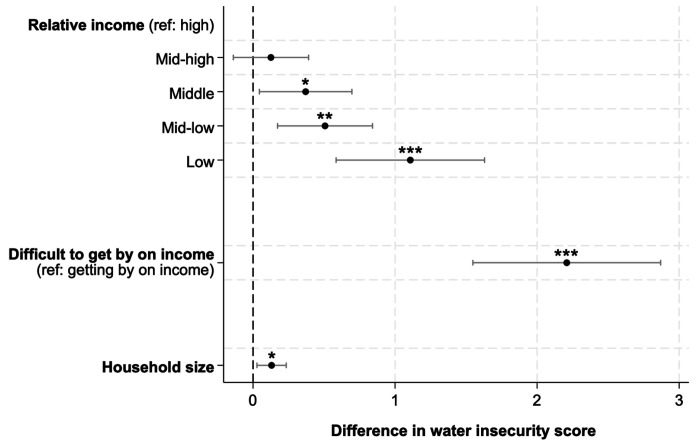
Variation in IWISE scores by sociodemographic characteristics among participants in the United States and Australia (Gallup World Poll 2022, n=1,992).^a^ ^a^Coefficients are from three different models. Each model includes the exposure of interest and adjusts for site and the complex sampling strategy. * *p*<0.05; ** *p*<0.01; *** *p*<0.001

As hypothesized, individuals with lower incomes reported greater experiences of water insecurity (Fig. 2). In multivariable models that adjusted for site and accounted for the complex sampling design, participants in the lowest income quintile were estimated to score 1.11 points higher (95% CI: 0.58, 1.63) on the IWISE Scale than those in the highest income quintile (Supplementary Table 9). Similarly, those reporting difficulty getting by on their present income were estimated to score 2.22 points higher (95% CI: 1.55, 2.87) on the IWISE Scale than those reporting no difficulties (Supplementary Table 9). Larger household size was similarly associated with higher IWISE scores as hypothesized. Each additional household member was associated with scoring 0.13-points higher on the IWISE Scale (95% CI: 0.03, 0.23; Fig. 2; Supplementary Table 9).

Another characteristic of a valid scale is that scores covary with related outcomes in expected directions. We tested whether higher IWISE scores were associated with self-reported stress and dissatisfaction with water quality. As expected, the percentage of participants with moderate-to-high water insecurity reporting stress during the previous day or water quality dissatisfaction was 1.80 times (95% CI: 1.50, 2.17) and 4.12 times (95% CI: 2.87, 5.93) higher, respectively, compared to those with no-to-mild water insecurity (Table 5). Analyses using continuous IWISE scores yielded similar results, with significant associations in the expected directions (Table 5).

## DISCUSSION

In this investigation of the suitability of the IWISE Scale in HICs, we found that the tool performed well. In cognitive interviews, the items were similarly understood among adults across five sites in HICs, with no reported concerns about translation, interpretation, or response. Analyses of nationally representative survey data from the United States and Australia supported the internal consistency, unidimensionality, and measurement invariance of the scale. The IWISE Scale was also associated with economic status, household size, self-reported stress, and dissatisfaction with water quality in the directions hypothesized, providing evidence of construct validity.

Establishing the appropriateness of the WISE Scales for use in HICs is timely and important. These tools are increasingly being integrated into monitoring efforts globally, and their application in HICs is expanding (42). The first published use of a WISE Scale in a HIC was in 2019–2020, when the HWISE Scale was administered among Six Nations households in Ontario, Canada (31). In 2022, it was also implemented in Walgett, Australia, a predominantly Aboriginal town (24, 25). That same year, the IWISE Scale was implemented in the United States and Australia via the Gallup World Poll, marking the first nationally representative WISE data to be collected in HICs (44). Currently, site-specific WISE data collection is ongoing in and around Chicago, Illinois, and in several Indigenous communities in the United States.

Findings from this study align with the limited published evidence on the use of the WISE Scales in HICs. In Canada, the HWISE Scale was found to have high internal consistency (Cronbach’s alpha = 0.815) among Six Nations households, and higher scores were correlated with lower household water access, community water access, and water infrastructure access (31, cf. Supplementary Material 3). In Walgett, Australia, higher HWISE scores were associated with related outcomes in expected directions, including greater household food insecurity (24).

These results support the continued use and expanded adoption of the WISE Scales for global monitoring. Experiential measures have proven valuable for capturing critical and actionable information on resource insecurities, including food, energy, and housing (56–58). Their added value is evidenced by the adoption of the Food Insecurity Experiences Scale as an indicator for Sustainable Development Goal Target 2.1 (59). Importantly, these measures are not limited to use for global monitoring (60). In the case of food insecurity, such tools have also been used to evaluate the impact of interventions, guide policy and program implementation, and support clinical screening and care (61, 62).

The WISE Scales are intentionally limited to universal experiences that have demonstrated relevance across diverse settings. Additional items, such as those related to water affordability, may be important in certain contexts but not globally applicable. For example, households may not routinely pay for water services, including in some HICs like the Republic of Ireland (63). We therefore recommend that users include the full WISE Scale that is appropriate for their needs (i.e., the individual or the household version) and supplement it with additional items tailored to local conditions or research priorities; this will ensure comparability. The WISE Manual provides guidance on context-specific adaptations and suggests optional items that can be used alongside the core scale (55). Many more items are, of course, possible.

As demonstrated in this study, each item in the IWISE Scale contributes meaningfully to the measurement of water insecurity. Retaining the full validated scale is thus essential for making comparisons within and across countries. Moreover, including these items enables findings to be situated within the growing body of nationally representative data available from multiple countries (44, Fig. 1). For example, in Walgett, Australia, the prevalence of moderate-to-high water insecurity was estimated to be 44% (64), which was much higher than the national prevalence of less than 1% reported by the Gallup World Poll. Documentation of this inequity helped prompt a coordinated government response to address local water issues (65).

This study has several strengths, including its mixed-methods design, use of cognitive interviews across diverse HIC settings, and rigorous psychometric evaluation using nationally representative survey data. Nonetheless, there are several limitations. First, the quantitative analyses included only two HICs, which may limit generalizability. Second, we were unable to assess test-retest reliability. Third, this study evaluated only the IWISE Scale. Although prior research in LMICs has found that the HWISE and IWISE Scales perform similarly well, the HWISE Scale has yet to rigorously evaluated in a HIC. Going forward, we encourage others who implement the WISE Scales in HICs to evaluate their reliability and validity using the procedures described herein. Continued testing will help ensure that these tools remain meaningful and comparable across diverse contexts.

## CONCLUSION

The WISE Scales accurately and equivalently measure the construct of water insecurity across low-, middle-, and high-income countries. By capturing user-side experiences, they provide critical insights that complement traditional provider-side indicators (66), and can help reveal problems related to water access and use that might otherwise go unrecognized (41, 52). To date, research using the WISE Scales has found greater water insecurity to be associated with poorer physical (6, 67, 68) and mental (69–71) health, greater food insecurity and malnutrition (9, 26, 29, 72), increased risk of violence (73, 74), and reduced economic well-being (55, 75). As such, the WISE Scales are valuable tools for advancing a more comprehensive understanding of water insecurity and for informing policies and programs that support equitable and reliable access to water worldwide.

## Supplementary Material

This is a list of supplementary files associated with this preprint. Click to download.

HICValidOSMv3.docx

## Figures and Tables

**Table 1:** Summary of participants in cognitive interviews about the IWISE Scale across five sites in four high-income countries (n = 73).

Sampling region	Site investigator	Interview dates	Language	Sample size (n women)	Age range
Flint, Michigan, USA	Pauli	October–November 2020	English	15 (9)	18–66
Throughout the United Kingdom	Staddon	October–November 2020	English	20 (12)	22–58
North Brabant, South Holland, and Utrecht, Netherlands	Vonk	October–November 2020	Dutch	15 (8)	18–55
Throughout Bulgaria	Staddon	November–December 2020	Bulgarian	16 (8)	23–89
New Orleans, Louisiana, USA	Collins	June 2023	English	7 (5)	18–55

**Table 2:** Sociodemographic characteristics of nationally representative samples from five Gallup World Poll countries used to establish the suitability of the IWISE Scale in high-income countries.^a^

	USA	Australia	Bangladesh	Brazil	Uganda
	(n = 1,001)	(n = 991)	(n = 1,007)	(n = 990)	(n = 939)
**Gender**, %					
Men	48.7	49.4	51.0	48.1	46.8
Women	51.3	50.6	49.0	51.9	53.2
**Age (years)**, mean ± SD	47.0 ± 19.2	48.3 ± 19.2	32.9 ± 12.6	38.7 ± 17.0	30.0 ± 10.4
**Education**, %					
Elementary	4.9	3.4	31.0	28.4	32.4
Secondary	61.7	69.4	60.3	61.4	66.8
Tertiary	33.4	27.2	8.6	3.4	0.9
**Marital status**, %					
Never married	36.1	27.0	32.1	41.1	50.5
Married or domestic partnership	45.9	54.7	66.3	48.8	38.6
Divorced, separated, or widowed	18.0	18.3	1.6	10.1	10.8
**Difficulty getting by on present income**, %	18.1	12.5	32.1	31.1	76.6
**IWISE score**, median (IQR)	0 (0–1)	0 (0–1)	0 (0–0)	2 (0–7)	7 (1–13)
**Water insecurity level**, %					
No-to-low	84.4	90.0	86.0	59.1	29.8
Mild	11.9	9.1	4.7	24.5	38.1
Moderate	3.0	1.0	5.1	13.1	25.6
High	0.7	0.0	4.2	3.4	6.5

a Estimates calculated using survey weights

**Table 3:** Internal consistency, factor loadings, and dimensionality of IWISE data across five countries in the Gallup World Poll, by country income level.

	USA	Australia	Bangladesh	Brazil	Uganda
	(n = 1,001)	(n = 991)	(n = 1,007)	(n = 990)	(n = 939)
**Internal consistency**					
Cronbach’s alpha (> 0.80)	0.907	0.837	0.963	0.894	0.912
**Standardized factor loadings (> 0.70)**					
Worry	0.751	0.758	0.956	0.786	0.722
Interruptions	0.839	0.804	0.936	0.847	0.707
Clothing	0.919	0.866	0.979	0.897	0.786
Plans	0.883	0.793	0.990	0.879	0.803
Food	0.863	0.872	0.964	0.846	0.793
Hands	0.865	0.835	0.972	0.799	0.805
Body	0.931	0.905	0.978	0.803	0.840
Drink	0.862	0.832	0.968	0.717	0.821
Angry	0.902	0.822	0.956	0.874	0.779
Sleep	0.912	0.929	0.954	0.793	0.819
None	0.842	0.834	0.920	0.795	0.828
Shame	0.893	0.716	0.925	0.829	0.760
**Model fit** ^a^					
RMSEA (< 0.06)	0.045	0.031	0.048	0.063	0.074
Upper RMSEA 90% CI (< 0.06)	0.053	0.040	0.056	0.070	0.082
CFI (> 0.95)	0.986	0.987	0.998	0.985	0.976
TLI (> 0.95)	0.983	0.984	0.997	0.981	0.971
SRMR (< 0.08)	0.041	0.065	0.027	0.054	0.036

a Values in parentheses indicate suggested parameters; red shading indicates estimates outside the parameter.

RMSEA: root mean square error of approximation; CI: confidence interval; CFI: comparative fit index; TLI: Tucker–Lewis index; SRMR: standardized root mean square residual

**Table 4:** Measurement invariance of aggregated IWISE data across five countries in the Gallup World Poll (n = 4,928). ^a^

	Configural invariance	Scalar invariance
**RMSEA (< 0.06)**	0.051	0.058
**Upper RMSEA 90% CI (< 0.06)**	0.055	0.061
**CFI (> 0.95)**	0.994	0.988
**TLI (> 0.95)**	0.992	0.990
**SRMR (< 0.08)**	0.047	0.056

a Values in parentheses indicate suggested parameters; red shading indicates estimates outside the parameter.

RMSEA: root mean square error of approximation; CI: confidence interval; CFI: comparative fit index; TLI: Tucker-Lewis index; SRMR: standardized root mean square residual

**Table 5:** IWISE scores were associated with greater stress and dissatisfaction with water quality in the United States and Australia (Gallup World Poll 2022, n = 1,992).^a^

	Experienced stress yesterday			Dissatisfied with water quality		
	PR	95% CI	*p*	PR	95% CI	*p*
**IWISE score** (continuous)	1.04	(1.03, 1.05)	< 0.001	1.09	(1.06, 1.11)	< 0.001
**Moderate-to-severe water insecurity** (ref: no-to-mild)	1.80	(1.50, 2.17)	< 0.001	4.12	(2.87, 5.93)	< 0.001

a Each model includes the exposure of interest and adjusts for site and the complex sampling strategy.

PR: prevalence ratio; CI: confidence interval

## Data Availability

The datasets used for the current study are available from the corresponding author on reasonable request.

## Abbreviations

CFI: comparative fit index
CI: confidence interval
HICs: high-income countries
HWISE: Household Water Insecurity Experiences
IWISE: Individual Water Insecurity Experiences
LMICs: low- and middle-income countries
PR: prevalence ratio
RMSEA: root mean square error of approximation
SRMR: standardized root mean square residual
TLI: Tucker-Lewis index
WISE: Water Insecurity Experiences
WLSMV: weighted least square mean- and variance-adjusted
